# Liquid AI for embodied robotics: From brittle black-box policies to adaptive and traceable control

**DOI:** 10.1016/j.xinn.2026.101542

**Published:** 2026-08-22

**Authors:** Kaiming Liu, Gongyi Zhu, Zihao Liu, Minyan Ge, Zice Wang, Shumao Xu

**Affiliations:** 1 Institute of Science and Technology for Brain-inspired Intelligence (ISTBI), Fudan University, Shanghai 200040, China; 2 Max Planck Institute, 70569 Stuttgart, Germany

## Main text

Embodied robotics is moving from controlled benchmarks into open, changing, and safety-critical environments. In these settings, perception, state estimation, and action are coupled through the robot’s motion: an early error in attention, depth estimation, or target tracking can alter the next observation and accumulate into unstable control, trajectory drift, or unsafe contact. Liquid artificial intelligence (liquid AI) is relevant to this challenge because it treats control-relevant computation as an evolving state process rather than a fixed mapping from isolated sensory frames to actions. Here, liquid AI is used as an umbrella term for compact neural controllers whose computation depends on evolving internal states, often through continuous-time or state-dependent dynamics and sparse recurrent structure. Within this framework, liquid time-constant (LTC) networks define input- and state-dependent continuous-time dynamics, closed-form continuous-time (CfC) networks provide a solver-free analytic realization of liquid-style state evolution, and neural circuit policies (NCPs) specify sparse, biologically inspired wiring that can be combined with these dynamical formulations. Existing evidence is strongest for compact controllers in constrained embodied tasks, including lane keeping and out-of-distribution visual navigation, whereas broad autonomy, long-horizon reasoning, multimodal manipulation, and formal safety assurance remain insufficiently established. Liquid models are therefore best viewed as candidate components for local perception-state-control loops that require rapid state updating, low-latency onboard execution, and traceable signal flow (Figure 1).

**Figure 1:**
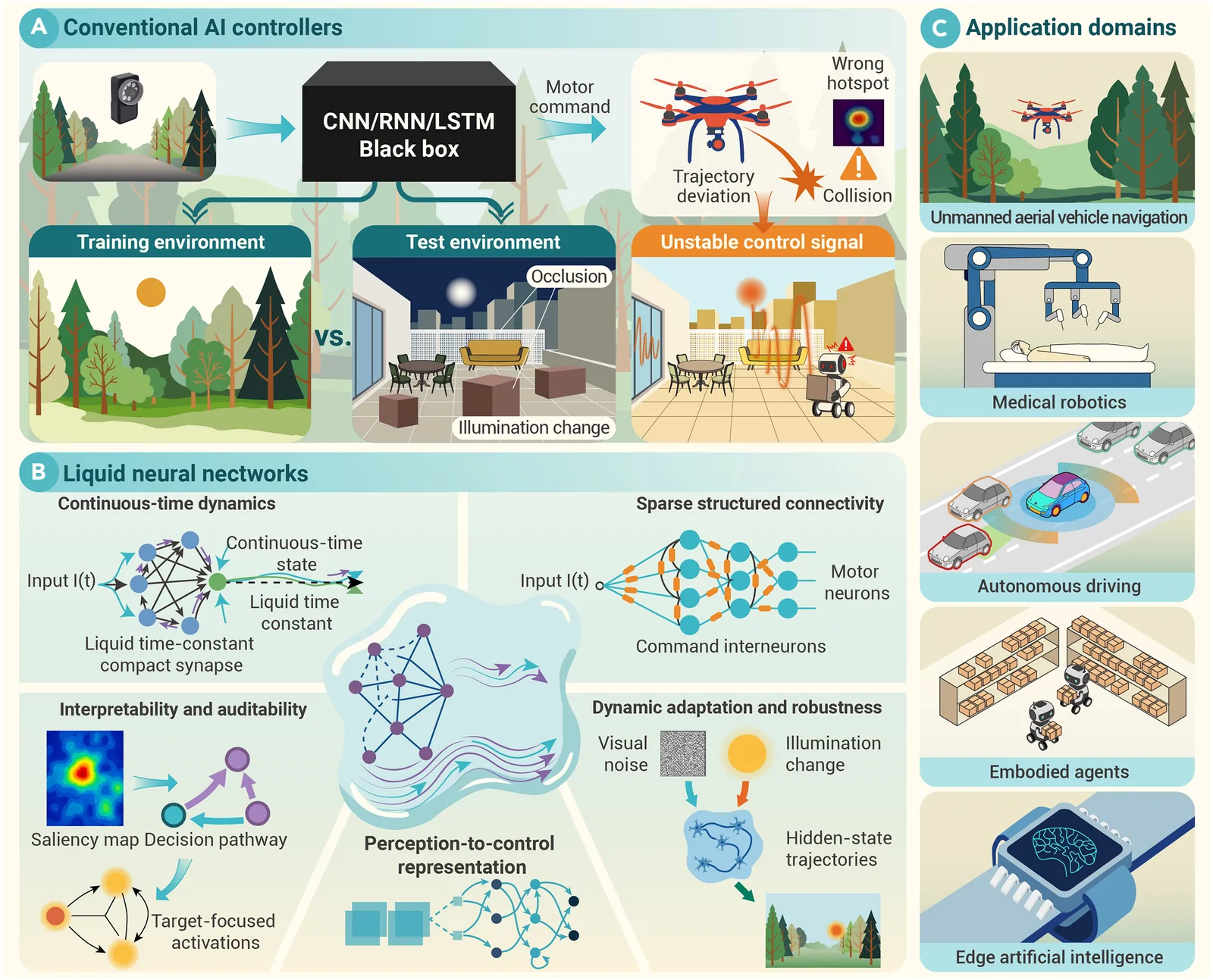
Liquid AI in embodied robotic control (A) Distribution shift can destabilize closed-loop behavior. (B) Liquid models combine continuous-time state dynamics, sparse wiring, hidden-state adaptation, and traceable signal flow. (C) Potential applications include UAV navigation, autonomous driving, embodied agents, medical robotics, and edge AI.

## From benchmark accuracy to closed-loop robustness

Robot learning is being shaped by two complementary pressures. One is scale: vision-language-action models suggest that semantic and task-level knowledge learned from large multimodal corpora can be transferred into robotic action. The other is state: recent state-space models have revived interest in efficient latent dynamics for long-range sequence modeling.^1^ These advances broaden what robotic policies can perceive, remember, and condition on, but they do not resolve a central control problem: which internal state is sufficient for safe action, how it should be updated under uncertainty, and how the policy behaves when environmental dynamics depart from training. Figure 1A illustrates this problem at the level of closed-loop robustness. In a physical robot, an error in visual attention, pose estimation, or temporal filtering changes the motor command; that command alters the robot’s motion and next observation, which is then processed again by the same policy. Through this feedback loop, a modest sensory shift can accumulate into trajectory drift or unsafe contact. Adaptive control, robust control, and model predictive control (MPC) therefore remain important comparators because, when system dynamics, constraints, or uncertainty sets can be modeled with sufficient fidelity, they provide formal tools for analyzing stability, constraint satisfaction, and closed-loop safety.

Liquid models address a narrower set of subproblems within short-timescale perception-state-control loops, including filtering asynchronous sensory streams, estimating target or ego-motion, maintaining a compact control state under transient disturbances, and generating recurrent actions on embedded hardware. In a modular robot stack, a vision-language-action model can provide goals and semantic context, a transformer or state-space model can represent longer temporal context, and MPC can enforce constraints, while a liquid module supports rapid local state updating and control. Its value should be judged against strong baselines under matched data, compute, latency, and closed-loop disturbances.

## Continuous-time state dynamics and adaptive inference

A useful starting point for understanding liquid AI is the controller’s state equation: what variables are carried forward, how sensory input and elapsed time reshape them, and how the updated state is converted into action. This places liquid models within the broader lineage of continuous-time neural computation. Neural ordinary differential equation models (neural ODEs) and ODE-based recurrent neural networks (ODE-RNNs) make hidden-state evolution between observations explicit. NCPs impose sparse, circuit-like connectivity from sensory units through interneurons to motor units, thereby making compact signal pathways easier to inspect.^2^ LTC networks introduce input- and state-dependent time constants, allowing the temporal response of a controller to change as conditions vary.^3^ CfC networks retain liquid-style temporal dynamics while replacing repeated numerical integration with an analytic update, thereby reducing solver overhead.^4^ These concepts are related but not interchangeable: circuit wiring, state dynamics, and update computation are distinct design axes that can be combined. Figure 1B summarizes the corresponding design principles: continuous-time dynamics, sparse connectivity, structured perception-to-control representations, inference-time hidden-state adaptation, local robustness, and traceable signal flow. In this context, adaptation refers primarily to inference-time state evolution. In response to glare, occlusion, target displacement, or a transient sensor perturbation, a liquid controller can shift its hidden trajectory and settle toward a new local control response without changing its learned parameters. This is distinct from online learning, which updates weights during deployment, and from policy-level behavioral adaptation, which may require persistent memory, goal inference, planning, or world models. Claims about online parameter updating or autonomous strategy change therefore require separate mechanisms, safeguards, and experimental validation. These distinctions also clarify the relevant comparisons. Convolutional neural network (CNN)-based policies can extract powerful spatial features, but the temporal state needed for physical control should be supplied through recurrence, filtering, memory, or an external estimator. Long short-term memory (LSTM) networks and gated recurrent units provide discrete-time recurrence and remain strong baselines when observations arrive at regular intervals, although their updates are tied to sequence steps rather than explicitly to elapsed physical time. Neural ODEs and ODE-RNNs make continuous-time evolution explicit, but their deployment behavior depends on solver choice and numerical discretization. State-space models are designed for scalable latent-state processing over long sequences, whereas liquid controllers are typically smaller dynamical systems intended for fast sensorimotor loops. Liquid models should therefore be evaluated where irregular sensing, elapsed time, recurrent state evolution, and embedded execution are central rather than where the primary challenge is open-ended semantic reasoning.

## Evidence, boundaries, and deployment safety

Current evidence supports a bounded but meaningful claim. Visual lane keeping with NCPs showed that sparse wiring can support compact policies and inspection of sensor-to-motor pathways,^2^ while liquid-network-based unmanned aerial vehicle (UAV) navigation was evaluated under out-of-distribution visual conditions involving changes in background, season, target appearance, and occlusion^5^ (Figure 1C). LTC and CfC studies further establish the expressive and computational value of continuous-time sequence modeling,^3^^,^^4^ although these findings are better interpreted as evidence for adaptive local dynamics than for general-purpose robot autonomy. Most demonstrations still use restricted visual inputs or low-dimensional control states and do not encompass long-horizon manipulation, clinical robot control, human-robot interaction, or rare safety-critical failures.

This evidentiary boundary matters when discussing interpretability. Sparse wiring, hidden-state trajectories, saliency maps, and active pathways can support debugging and post hoc analysis. In UAV navigation, for example, investigators can examine whether occlusion changes the hidden-state trajectory, activates a particular pathway, and corresponds to a steering response.^5^ This analysis can indicate whether the controller tracks task-relevant motion or responds to a spurious visual hotspot, but it does not establish closed-loop safety. Traceability means that internal states or signal pathways can be inspected and linked to an observed action, whereas interpretability requires a human-meaningful account of why that action occurred. Auditability further requires documented and reproducible evidence that can be independently reviewed, while formal verification asks whether specified initial states, disturbances, sensor faults, or actuator limits can lead to violation of a defined safety property.

The remaining challenges span training stability, numerical sensitivity, verification, and systems integration. In LTC-style models, time-constant initialization can produce sluggish or overly reactive dynamics, while discretization choices and solver tolerances can affect both accuracy and computational cost. Training can also remain sensitive to exploding or vanishing gradients and to hyperparameter choices. CfC-style analytic updates reduce solver overhead, but they do not remove the need for matched baselines, ablation of gates and time constants, or closed-loop failure analysis. Because liquid dynamics are nonlinear, input dependent, and coupled to uncertain perception modules, deployment should incorporate runtime monitoring, fallback control, safety envelopes, or reachability-informed checks.

Long-horizon autonomy and hardware deployment introduce additional limitations. Liquid states may stabilize local perception-state-control loops but do not provide persistent task memory, counterfactual reasoning, hierarchical planning, or language-conditioned goal management. Likewise, deployment claims require measurements on the target hardware, including end-to-end latency, memory traffic, power consumption, numerical-precision sensitivity, and recovery after transient failure; parameter count alone is insufficient.

Liquid AI is most credible as a family of learned continuous-state controllers for fast local loops within a layered robotic system. Its contribution should be evaluated through matched closed-loop comparisons, systematic assessment of performance degradation under specified distribution shifts, transparent reporting of hyperparameters and failure modes, and integration of traceable state trajectories with runtime monitoring or formal safety arguments.

## Funding and acknowledgments

This work was supported by the 10.13039/501100001809National Natural Science Foundation of China (22205254) and the 10.13039/501100008838Shanghai Municipal Commission of Economy and Informatization through the Science Intelligence “Hundred Teams, Hundred Projects” Program (CWH0082502). We further acknowledge the CFFF High-Performance Computing Platform at Fudan University for providing computational resources. The authors thank the editors and reviewers for their invaluable comments, which helped improve this work.

## Declaration of interests

The authors declare no competing interests.
